# Seasonal variations of the relationships between measures of training monotony and strain in professional soccer players

**DOI:** 10.1038/s41598-022-15278-4

**Published:** 2022-06-29

**Authors:** Hadi Nobari, Alexandre Duarte Martins, Rafael Oliveira, Luca Paolo Ardigò

**Affiliations:** 1 Department of Exercise Physiology, Faculty of Educational Sciences and Psychology, University of Mohaghegh Ardabili, Ardabil, Iran; 2 Department of Motor Performance, Faculty of Physical Education and Mountain Sports, Transilvania University of Braşov, 500068 Brasov, Romania; 3 Faculty of Sport Sciences, University of Extremadura, 10003 Cáceres, Spain; 4 Sepahan Football Club, Isfahan, 81887-78473 Iran; 5 Sports Science School of Rio Maior–Polytechnic Institute of Santarém, 2040-413 Rio Maior, Portugal; 6 Life Quality Research Centre, 2040-413 Rio Maior, Portugal; 7 Departamento de Desporto e Saúde, Comprehensive Health Research Centre (CHRC), Escola de Saúde e Desenvolvimento Humano, Universidade de Évora, Largo dos Colegiais, 7000-727 Évora, Portugal; 8 Research Center in Sport Sciences, Health Sciences and Human Development, 5001-801 Vila Real, Portugal; 9 Department of Neurosciences, Biomedicine and Movement Sciences, School of Exercise and Sport Science, University of Verona, 37131 Verona, Italy

**Keywords:** Medical research, Engineering, Materials science

## Abstract

The purposes of this study were (a) to determine the variations in internal and external measures of training monotony (TM) and strain (TS) in professional soccer players according to periods of the season and playing positions, and (b) to analyze the relationships between internal and external measures of TM and TS. Twenty male professional players (age = 29.4 ± 4.4 years) were followed for 20 weeks through session rating of perceived exertion (s-RPE), total distance (TD), high-speed running distance (HSRD) and sprint distance (SpD). Regardless of measure, highest mean TM and TS scores were observed in mid-season and end-season. In general, wingers and strikers tended to have greater values in TM. Midfielders exhibited greater TS of TD and SpD. Correlation results for TM revealed that s-RPE was positively associated with SpD in early-season (r = 0.608) and negatively associated in mid-season (r = − 0.506). Regarding the TS, result demonstrated that s-RPE is negatively associated with HSRD in early-season (r = − 0.464) and positively associated in mid-season (r = 0.476). In general, there different meanings in correlations between internal and external measures across the season. On the one hand, our findings highlighted that TM and TS of professional soccer players is sensitive to period of the season and player’s position, but on other hand, correlation analyses proved that changes in one external/internal measure does not cause changes in another external/internal measure which support the constant monitoring of these values across the season.

## Introduction

The knowledge of load dynamics in soccer would help coaches, their staff, and practitioners to improve performance and at the same time to avoid fatigue, injury and illness^1^. In addition, it is well known that load measures may vary from session to session^2–4^ week to week^5–8^, mesocycle to mesocycle^2,5,9^ and/or period to period^4,6,10–13^.

Some indexes that allow to interpret load variations are known as training monotony (TM) and training strain (TS)^14^. While TM is calculated through the daily mean load divided by the week standard deviation, TS is based on TM multiplied by the accumulated load of the week^15^.

Another major factor that influences load dynamic is the positions of the players. Previous studies reported that player positions have different physical roles and consequently different load during matches^16–20^. Such differences were also revealed in training^2,4,21^ and recently it was shown that external defenders and wingers presented greater TS for high-speed running and number of sprints during the season compared to the remaining positions. However, another study found no significant differences between positions for TM and TS calculated through decelerations, accelerations, impacts and high metabolic load distance^10^. Thus, more research on those metrics and between player positions is needed to confirm or not the results of the previous studies.

Beyond the information given before about TM and TS, some studies calculated both indexes through running distance variables^5–7^ and through s-RPE variable^5,7,9,22^. The study of Oliveira et al.^5^ included both external and internal workload measures in simultaneously but failed to analyze them taking into account the player positions while the other study of Oliveira et al.^7^ seems to be the only one that **analyzed** both external and internal workload measures considering player positions. Specifically, this study found significant differences between player positions with moderate to very large effect across 10 mesocycles of the in-season. However, the previous study had small sample size and recommended more research on this topic. Moreover, the differences in the periods analyzed (10 mesocycles) reinforced that more analysis could be performed considering different periods of the season (e.g. pre-season and in-season). Furthermore, none of the previous studies^5–7,9^ showed the relationships between TM and TS calculated through internal and external measures.

The relationship between internal and external load measures have been analyzed in previous studies^23–25^ although without considering TM and TS indexes. Specifically, a study with professional soccer players showed that rating perceived exertion (RPE) correlate with distances covered between 14.4 and 19.8 and between 19.9 and 25.1 km/h^23^. Another study with professional soccer players also found a relationship between session-RPE (s-RPE) and total distance and between s-RPE and distances covered at > 19.8 km/h^24^. Such findings were also confirmed in young soccer players^25^.

Therefore, the aim of this study was (a) to describe and compare the in-season variations of TM and TS through s-RPE, total distance (TD), high-speed running distance (HSRD) and sprint distance (SpD) across different periods of a professional soccer season (early-season, mid-season, and end-season) and according to player positions (defenders, midfielders, wingers and strikers), and (b) to analyze the relationship of the aforementioned internal with external workload indexes measures across different periods of the season, respectively.

## Methods

### Participants

Twenty professional players from an Asian First League (29.4 ± 4.4 years old; 75.0 ± 3.9 kg; 1.8 ± 0.1 cm; BMI: 23.4 ± 1.8 kg/m^2^) participated in this study. Five players from each position were selected from the entire number of participants, including defenders (DF), midfielders (MF), wingers (WG), and strikers (SF). It were included only players, who (1) were part of the team from week 1 to week 20 and (2) participated in 80% of weekly training sessions. It were not included players (1) with prolonged injury or a lack of participation in training for at least two consecutive weeks, (2) who showed the initial physical fitness test scores two standard deviations below the squad mean and (3) whose position was goal keeper due to differences in training activities and workload in training and matches. At the very beginning of the research, the players were informed about the study design and procedures. Thereafter, the players signed a free consent about their participation in the study. They did it even if this research’s methods were already part of their club daily routine. This research fulfilled the requirements of the Declaration of Helsinki under the approval of the Ardabil University of Medical Sciences research ethics committee.

### Experimental design

This research makes use of a descriptive-longitudinal approach. Players’ monitoring occurred over 20 consecutive in-season weeks. All team’s main training sessions were part of this research. Rehabilitation and recuperation sessions were not taken into account. Training sessions were made of warm-up, main and slow-down phases in addition to stretching. Coaching staff designed all training sessions, while researchers standardized only first and final 30 min (i.e., start and end of each session). Research took place from October 30, 2017 (early-season) until March 18, 2018 (end-season). Whole season was made of early-season (weeks 1–7), mid-season (weeks 8–13) and end-season (weeks 14–20; Table 1). Table 1 shows training sessions and matches numbers over the three season’s periods, as well.

**Table 1:** Weeks and training sessions and number of competitive matches.

Periods of the in-season	Early-season	Mid-season	End-season
Number of weeks	7	7	6
Training sessions (N)	15	14	18
Number of matches (N)	7	8	5

### External load monitoring

During each session, players were monitored by a GPS (GPSPORTS systems Pty Ltd, Model: SPI High-Performance Unit (HPU); Australian) and the study measures were collected daily during the in-season (i.e., all training sessions and matches). This study aimed to describe and compare the in-season variations of acute: TM and TS through s-RPE, total distance (TD), high-speed running distance (HSRD) and SpD across different periods of a professional soccer season (early-season, mid-season, and end-season) according to players’ positions.

Global navigation satellite systems for professional athletes, such as the SPI HPU, include a 15 Hz GPS sensor in addition to a tri-axial accelerometer. As already shown in the literature, used device shows high validity and reliability (Cohen’s *d* of differences between gold standard and device from trivial to small and intraclass correlation coefficients > 0.95)^26^. Throughout season, temperature and humidity resulted from 10 and 26 °C and from 22 to 48%, respectively. Special vests for the devices were placed on players’ shoulders before trainings and matches starts. After activities, devices were removed from the players and checked by the team’s match analyst before downloading recorded data to a computer equipped with the Team AMS software. Then, devices’ memories were “cleaned” from old data and devices were put on an electric re-charge station. Devices’ software was used according to manufacturer’s instructions including putting into it players' anthropometric information and personal vest’s assignment.

### Internal load monitoring

Players were daily monitored for their RPE using the CR-10 Borg’s scale^27^, adapted by Foster et al.^28^. Previous study demonstrated the validity and reliability of this scale to estimate the session intensity^29^. Thirty minutes after the end of each training session, players rated their RPE value using an app on a tablet. The scores provided by the players were also multiplied by the training duration, to obtain the s-RPE^28,30^. The players were previously familiarized with the scale, and all the answers were provided individually to avoid non-valid scores.

### Calculations of training indexes

Through s-RPE, TD, HSRD and SpD, the following measures were calculated: (1) TM (mean of training load during the 7 days of the week divided by the standard deviation of the training load of the 7 days^5–7,31^ and (2) TS (sum of the training load for all training sessions during a week multiplied by training monotony^5–7,31^.

### Statistical analysis

Descriptive statistics were used to characterize the sample. Shapiro–Wilk was used to test normality of results. Results were presented as mean ± standard deviation (SD). The relationship between all variables at the different periods was verified using bivariate correlations^32^ (Pearson product-moment correlation coefficient (*r*)). The effect size of the correlations was determined by considering the following thresholds: < 0.1 = trivial; 0.1–0.3 = small; > 0.3–0.5 = moderate; > 0.5–0.7 = large; > 0.7–0.9 = very large; and > 0.9 = nearly perfect^33,34^.

All measures obtained a normal distribution (Shapiro–Wilk > 0.05), it was used a repeated measures ANOVA test and the Bonferroni post-hoc test to compare measures for periods of the in-season and groups. The results are significant for a *p* ≤ 0.05. Hedge’s g effect size (ES) was also calculated to determine the magnitude of pairwise comparisons. The Hopkins threshold was utilized as follows: *g* ≤ 0.2, trivial; 0.2 < *g* ≤ 0.6, small; 0.6 < *g* ≤ 1.2, moderate; 1.2 < *g* ≤ 2.0, large; 2.0 < *g* ≤ 4.0, very large; and *g* > 4.0, nearly perfect^33^. All data were analysed using IBM SPSS Statistics (version 22, IBM Corporation (SPSS Inc., Chicago, IL).

### Ethics approval and consent to participants

To engage in this study, both the players and their staff coach signed an informed consent form. The study has approved by the Ardabil university of medical sciences Ethics Committee prior to its start, and the Helsinki Declaration was used to follow the recommendations of Human Ethics in Research.

## Results

**Figure 1:**
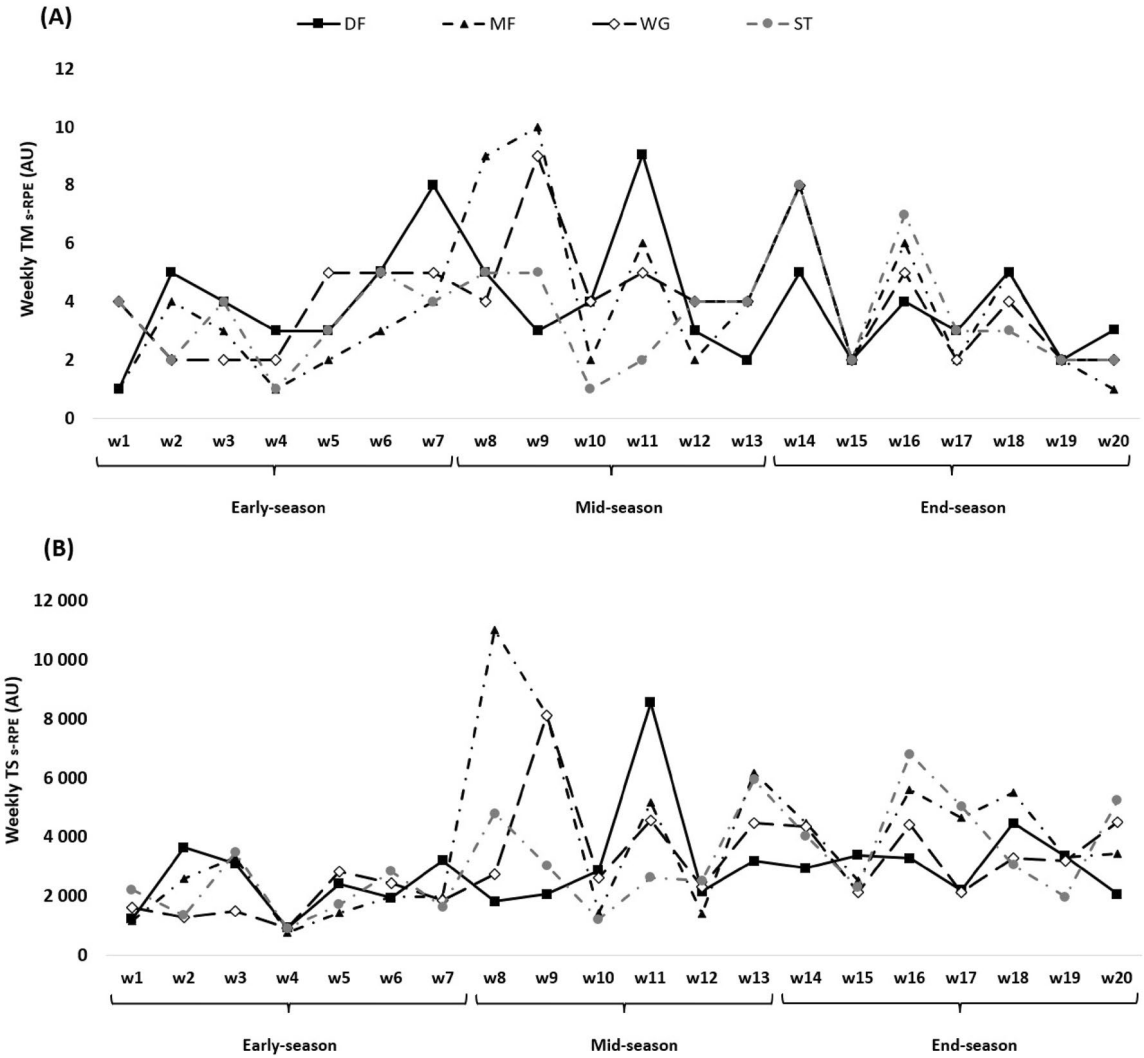
TM (**A**) and TS (**B**) variations calculated through the s-RPE across 20 weeks between players’ positions.

**Figure 2:**
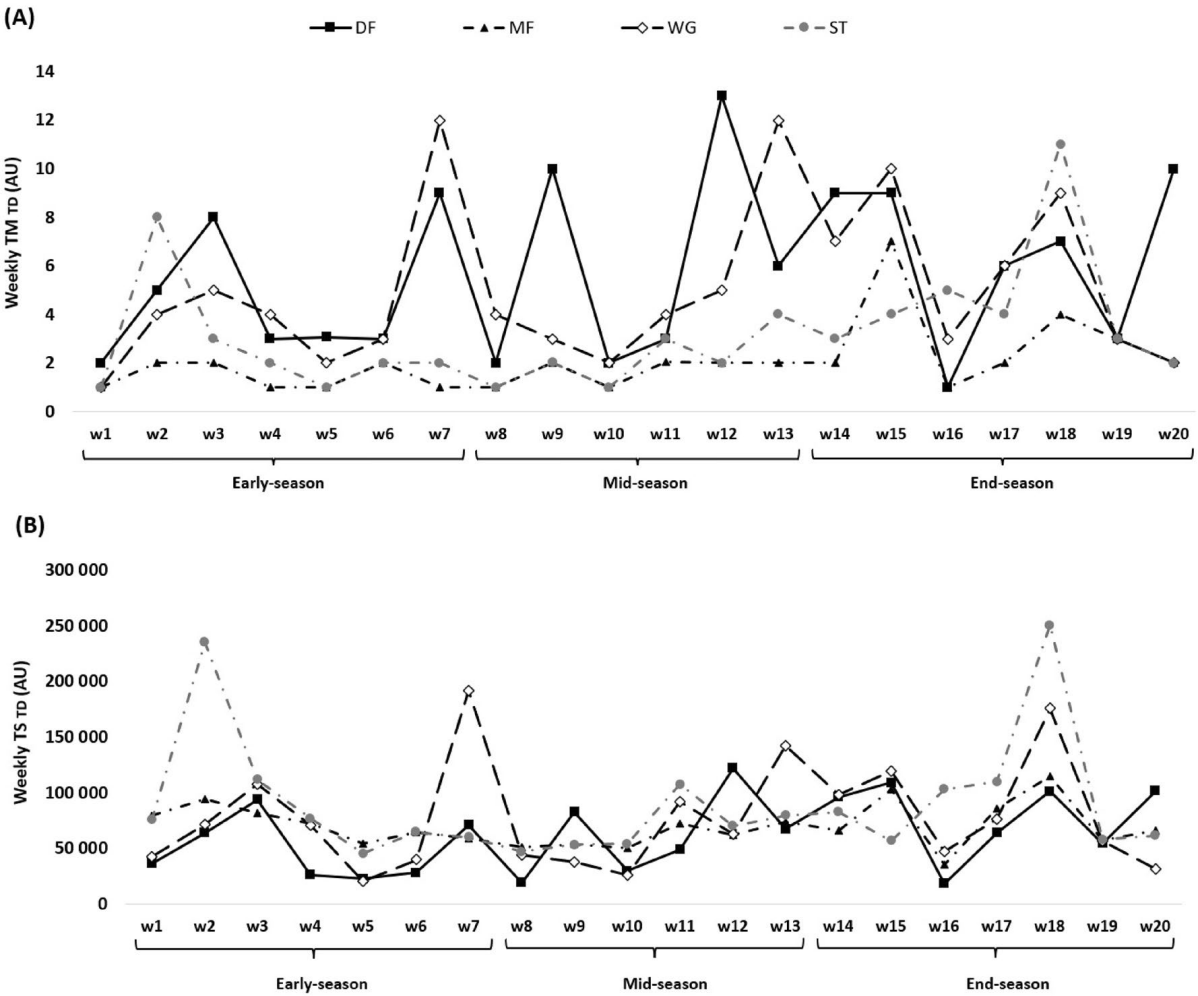
TM (**A**) and TS (**B**) variations calculated through the TD across 20 weeks between players’ positions.

**Figure 3:**
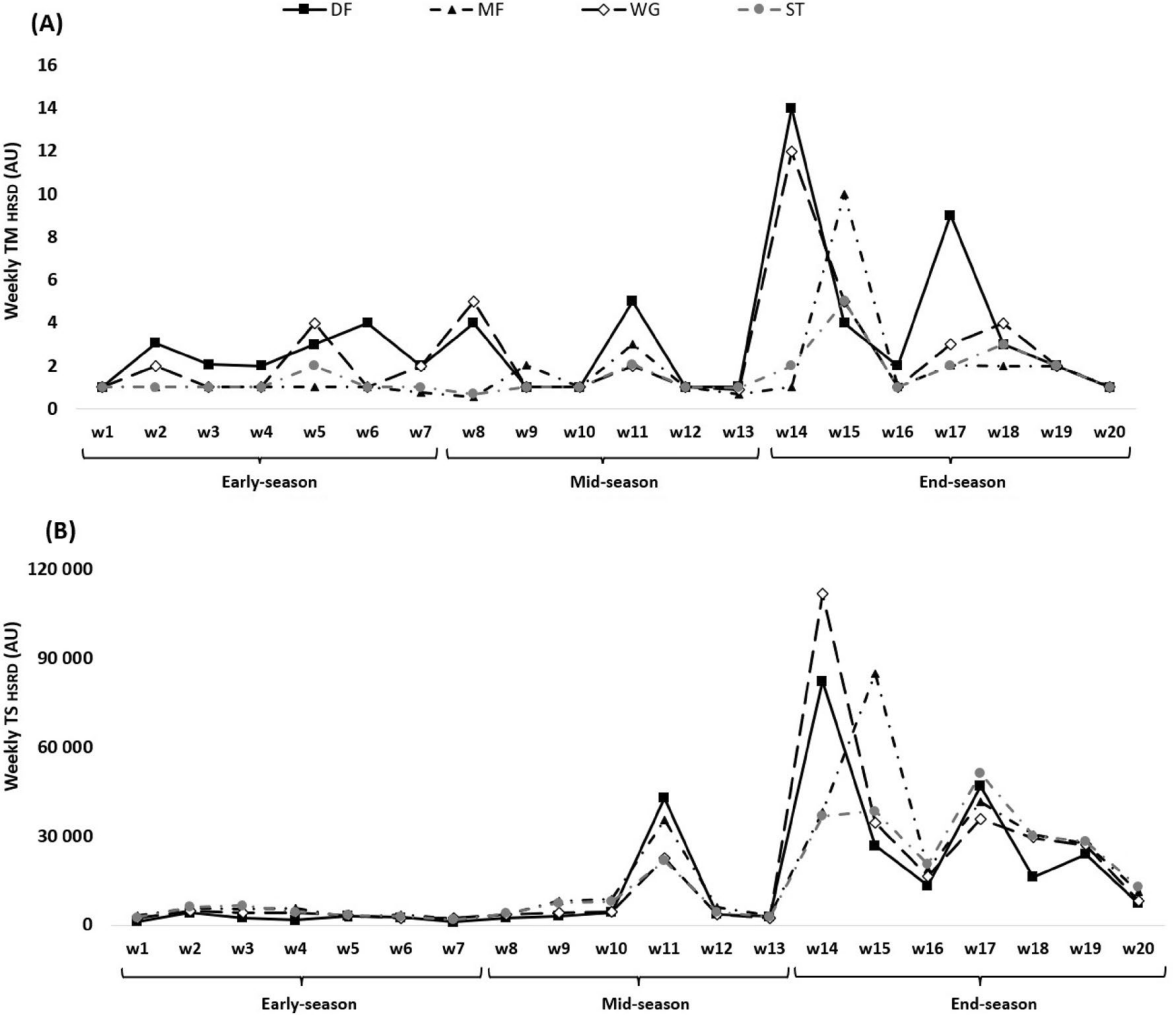
TM (**A**) and TS (**B**) variations calculated through the HSRD across 20 weeks between players’ positions.

**Figure 4:**
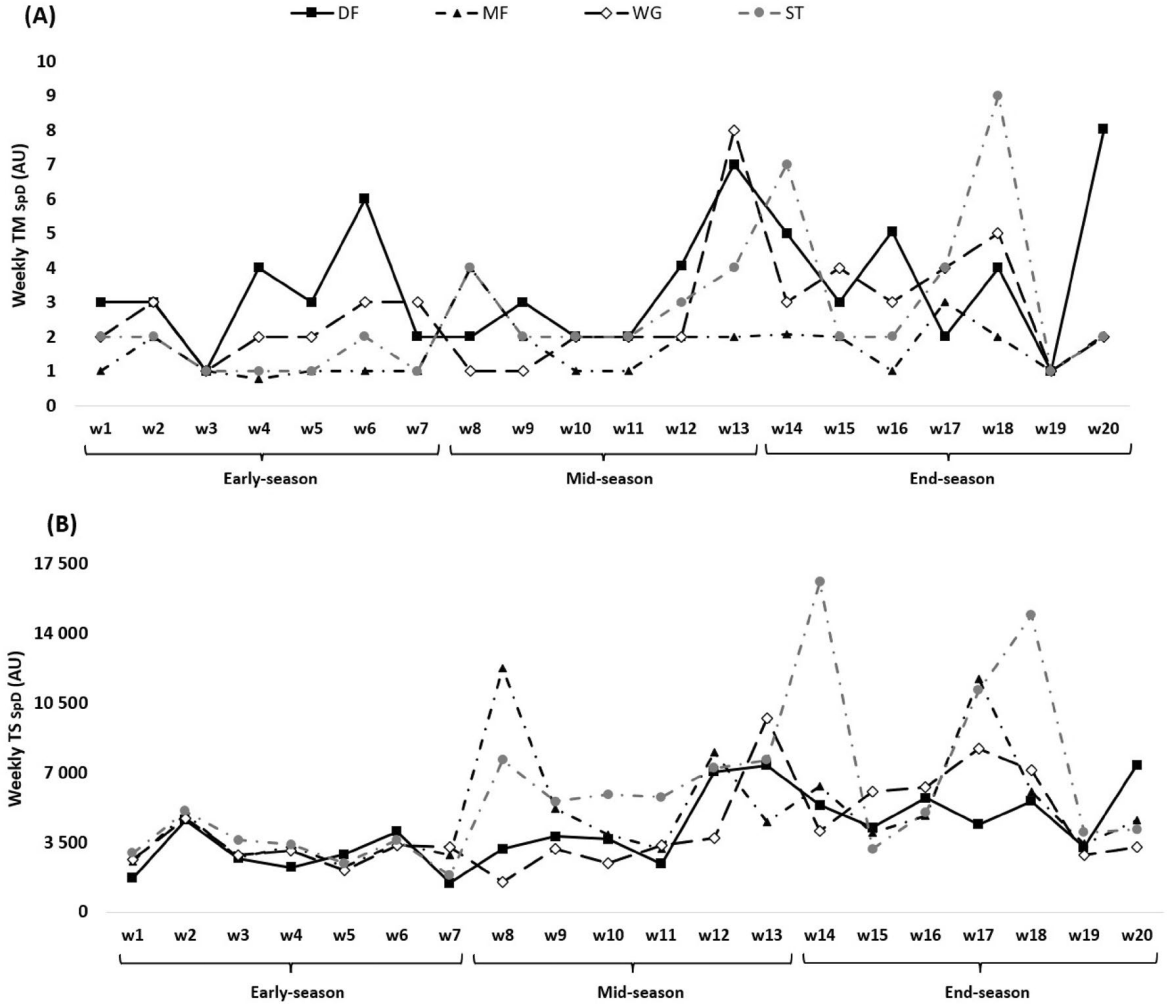
TM (**A**) and TS (**B**) variations calculated through the sprint distance across 20 weeks between players’ positions.

Figures 1, 2, 3 and 4 show an overall view of the weekly average for TM and TS calculated through s-RPE, TD, HSRD, and SpD across different periods of a professional soccer season (early-season, mid-season, and end-season) between players’ positions.

The weekly changes of TM and TS for s-RPE can be found in Fig. 1. The highest TM_s-RPE_ occurred in week 9 in mid-season (MF = 10.02 ± 3.00 arbitrary units (AU)) and the lowest values happened in week 1 in early-season (DF = 1.00 ± 0.01 AU and MF = 1.00 ± 0.01 AU), week 4 (early season) (MF = 1.00 ± 2.00 AU and ST = 1.00 ± 0.01 AU), week 10 (mid-season) (DF = 1.00 ± 0.01 AU) and week 20 in end-season (MF = 1.00 ± 0.01 AU). The TS_s-RPE_ was the highest in week 8 in mid-season (MF = 10,996.00 ± 6968.00 AU) and the lowest in in week 4 in early-season (MF = 10,996.00 ± 6968.00 AU).

The weekly changes of TM and TS for TD can be seen in Fig. 2. The highest TM_TD_ occurred in week 12 in mid-season (DF = 13.00 ± 11.00 AU) and the lowest values happened in week 1 in early-season (MF = 1.00 ± 0.02 AU, WG = 1.00 ± 0.01 AU and ST = 1.00 ± 0.01 AU), week4 (early season) (MF = 1.00 ± 0.01 AU), week 5 (early-season) (MF = 1.00 ± 0.01 AU and ST = 1.00 ± 0.01 AU), week 7 (early-season) (MF = 1.00 ± 0.01 AU), week 8 (mid-season) (MF = 1.00 ± 0.01 AU and ST = 1.00 ± 0.01 AU) and week 16 in end-season (DF = 1.00 ± 0.01 AU and MF = 1.00 ± 0.01 AU). TS_TD_ was the highest in week 18 in end-season (ST = 250,402.00 ± 346,684.00 AU) and the lowest in week 16 in end-season (DF = 18,423.00 ± 5765.00 AU).

The weekly changes of TM and TS for HSRD can be seen in Fig. 3. The highest TM_HSRD_ occurred in week 14 in end-season (DF = 14.00 ± 17.00 AU) and the lowest happened in week 8 in mid-season (MF = 0.55 ± 0.16 AU). The TS_HSRD_ was the highest in week 14 in end-season (WG = 111,872.00 ± 117,710.00 AU) and the lowest in week 7 in early-season (DF = 966.00 ± 647.00 AU).

The weekly changes of TM and TS SpD can be found in Fig. 4. The highest TM_SpD_ occurred in week 18 in end-season (ST = 9.00 ± 6.00 AU) and the lowest happened in week 4 in early-season (MF = 0.78 ± 0.18 AU). The TS_SpD_ was the highest in week 14 in end-season (ST = 16,580.00 ± 19,639.00 AU) and the lowest in week 7 in early-season (DF = 1444.00 ± 1580.00 AU).

Table 2 presents the differences between the early-season, mid-season, and end-season for TM and TS calculated through s-RPE, TD, HSRD, and SpD. To simplify the description, only large to nearly perfect effect sizes will be described here. There was no significant difference for TM_s-RPE_.

**Table 2:** Descriptive statistics (mean ± SD) of all measures in early-season, mid-season and end-season.

Measures	EarlyS (Mean ± SD)	MidS (Mean ± SD)	EndS (Mean ± SD)	*p*	Hedges’ g (95% CI)
TM_s-RPE_ (AU)	3.00 ± 0.10	5.00 ± 2.00	4.09 ± 0.01	EarS versus MidS: 0.059	–
				EarS versus EndS: 1.000	–
				MidS versus EndS: 0.077	–
TS_s-RPE_ (AU)	2007.09 ± 418.00	4119.00 ± 1884.00	3700.00 ± 806.00	**EarS versus MidS: 0.001**	− 1.52 [− 2.25, − 0.83]#
				**EarS versus EndS: < 0.01**	− 2.58 [− 3.48, − 1.77]§
				MidS versus EndS: 1.000	–
TM_TD_ (AU)	3.00 ± 2.00	4.00 ± 3.09	5.00 ± 2.00	EarS versus MidS: 1.000	–
				**EarS versus EndS: 0.010**	− 0.98 [− 1.65, − 0.33]*
				**MidS versus EndS: < 0.01**	− 0.38 [− 1.01, 0.24]&
TS_TD_ (AU)	73,645.00 ± 38,650.00	64,607.00 ± 22,224.00	85,703.00 ± 36,003.00	**EarS versus MidS: < 0.01**	0.28 [− 0.34, 0.91]&
				**EarS versus EndS: < 0.01**	− 0.32 [− 0.94, 0.30]&
				**MidS versus EndS: 0.032**	− 0.69 [− 1.34, − 0.06]&
TM_HSRD_ (AU)	2.01 ± 0.02	1.00 ± 0.10	4.02 ± 2.00	EarS versus MidS: 1.000	–
				**EarS versus EndS: < 0.01**	− 1.39 [− 2.11, − 0.72]#
				**MidS versus EndS: < 0.01**	− 2.09 [− 2.91, − 1.34]§
TS_HSRD_ (AU)	3402.00 ± 980.00	8866.00 ± 5909.00	33,956.00 ± 13,164.00	**EarS vesus MidS: 0.002**	− 1.26 [− 1.97, − 0.59]#
				**EarS versus EndS: < 0.01**	− 3.21 [− 4.22, − 2.30]§
				**MidS versus EndS: < 0.01**	− 2.41 [− 3.28, − 1.62]§
TM_SpD_ (AU)	2.00 ± 1.03	3.00 ± 1.02	3.00 ± 1.00	**EarS versus MidS: 0.011**	− 0.96 [− 1.63, − 0.31]*
				**EarS versus EndS: 0.003**	− 0.97 [− 1.64, − 0.32]*
				MidS versus EndS: 1.000	–
TS_SpD_ (AU)	3092.00 ± 609.00	5339.00 ± 1800.00	6201.00 ± 2498.00	**EarS versus MidS: < 0.01**	− 1.64 [− 2.39, − 0.94]#
				**EarS versus EndS: < 0.01**	− 1.68 [− 2.43, − 0.97]#
				**MidS versus EndS: < 0.01**	− 0.38 [− 1.02, 0.23]&

Significant differences between periods are highlighted in bold (*p* ≤ 0.05).

*AU* arbitrary units, *EarlyS* early-season, *MidS* mid-season, *EndS* end-season, *SD* standard deviation, *TM* training monotony, *TS* train strain, *s-RPE* session rate of perceived exertion, *TD* total distance, *HSRD* high‐speed running distance, SpD, sprint distance.

&small effect; *, moderate effect; #, large effect; §, very large effect; £, nearly perfect effect.

The TS_s-RPE_ presents a significant higher value in mid-season than early-season [large effect] and shows a significant higher value in end-season than early-season [very large effect].

The TM_HSRD_ presents a significant higher value in end-season than early-season [large effect] and shows a significant higher value in end-season than mid-season [very large effect]. The TS_HSRD_ shows a significant higher value in mid-season than early-season [large effect], shows a significant higher value in end-season than early-season [very large effect], and presents a significant higher value in end-season than mid-season [very large effect].

Finally, the TS_SpD_ presents a significant higher value in mid-season than early-season [large effect] and shows a significant higher value in end-season than early-season [large effect].

Table 3 presents the differences between player positions for TM and TS calculated through s-RPE, TD, HSRD, and SpD during in-season. There were no meaningful differences for TS_HSRD_. To simplify the description, only large to nearly perfect effect sizes will be described here.

**Table 3:** Descriptive statistics (mean ± SD) of all measures between players’ positions.

Measures	DF (Mean ± SD)	MF (Mean ± SD)	WG (Mean ± SD)	ST (Mean ± SD)	*p*	Hedges’ g (95% CI)
TM_s-RPE_ (AU)	4.00 ± 0.02	3.00 ± 0.01	4.00 ± 1.02	4.00 ± 0.58	DF versus MF: 1.000	−
					DF versus WG: 1.000	−
					DF versus ST: 1.000	–
					**MF versus WG: < 0.01**	− 1.25 [− 2.76, 0.05]#
					**MF versus ST: < 0.01**	− 2.20 [− 4.11, − 0.69]§
					WG versus ST: 1.000	−
TS_s-RPE_ (AU)	3943.00 ± 712.00	2990.00 ± 65.00	3062.00 ± 777.00	2937.00 ± 270.00	DF versus MF: 0.083	*–*
					**DF versus WG: < 0.01**	1.07 [− 0.21, 2.52]*
					DF versus ST: 0.061	−
					MF versus WG: 1.000	–
					MF versus ST: 1.000	–
					WG versus ST: 1.000	–
TM_TD_ (AU)	2.00 ± 0.01	3.00 ± 1.00	5.00 ± 1.00	6.00 ± 1.00	DF versus MF: 1.000	–
					**DF versus WG: 0.049**	− 3.83 [− 6.59, − 1.83]§
					**DF versus ST: 0.010**	− 5.11 [− 8.62, − 2.65]£
					**MF versus WG: < 0.01**	− 1.81 [− 3.53, − 0.39]#
					**MF versus ST: < 0.01**	− 0.90 [− 2.30, 0.36]&
					WG versus ST: 1.000	–
TS_TD_ (AU)	68,306.00 ± 6446.00	91,790.00 ± 28,738.06	77,714.00 ± 36,359.00	62,806.00 ± 14,552.00	**DF versus MF: < 0.01**	− 1.02 [− 2.45, 0.25]#
					DF versus WG: 1.000	−
					DF versus ST: 1.000	–
					MF versus WG: 1.000	–
					**MF versus ST: < 0.01**	1.15 [− 0.14, 2.62]*
					WG versus ST: 1.000	–
TM_HSRD_ (AU)	2.03 ± 0.01	1.00 ± 0.01	3.00 ± 1.00	3.00 ± 1.00	DF versus MF: 1.000	–
					**DF versus WG: < 0.01**	− 1.24 [− 2.74, 0.06]#
					**DF versus ST: < 0.01**	− 1.24 [− 2.74, 0.06]#
					**MF versus WG: < 0.01**	− 2.55 [− 4.63, − 0.95]§
					**MF versus ST: < 0.01**	− 2.55 [− 4.63, − 0.95]§
					WG versus ST: 1.000	–
TS_HSRD_ (AU)	17,612.00 ± 3265.00	14,354.00 ± 672.00	16,391.00 ± 6809.00	14,583.00 ± 6603.00	DF versus MF: 1.000	–
					DF versus WG: 1.000	–
					DF versus ST: 1.000	–
					MF versus WG: 1.000	–
					MF versus ST: 1.000	–
					WG versus ST: 1.000	–
TM_SpD_ (AU)	2.07 ± 0.08	3.00 ± 1.00	3.00 ± 0.22	3.00 ± 1.00	**DF versus MF: < 0.01**	− 1.18 [− 2.67, 0.11]*
					**DF versus WG: < 0.01**	− 5.07 [− 8.56, − 2.63]£
					**DF versus ST: 0.014**	− 1.18 [− 2.67, 0.11]*
					MF versus WG: 1.000	–
					MF versus ST: 1.000	–
					WG versus ST: 1.000	–
TS_SpD_ (AU)	5009.00 ± 390.00	6070.00 ± 1938.00	4190.00 ± 1014.00	4149.00 ± 705.00	DF versus MF: 1.000	–
					DF versus WG: 1.000	*–*
					DF versus ST: 1.000	–
					**MF versus WG: < 0.01**	− 1.09 [0.19, 2.56]*
					**MF versus ST: < 0.01**	1.16 [− 0.13, 2.64]*
					WG versus ST: 1.000	–

Significant differences between player positions are highlighted in bold (*p* ≤ 0.05).

*AU* arbitrary units, *DF* defenders, *MF* midfielders, *WG* wingers, *ST* strikers, *SD* standard deviation, *ACWR* acute: chronic workload ratio, *EWMA* exponentially weighted moving averages, *CP* coupled, *UCP* uncoupled, *s-RPE* session rate of perceived exertion, *TD* total distance, *HSRD* high‐speed running distance, *SpD* sprint distance.

&small effect; *, moderate effect; #, large effect; §, very large effect; £, nearly perfect effect.

The TM_s-RPE_ shows a significant higher value in WG than MF [large effect] and shows a significant higher value in ST than MF [very large effect].

The TM_TD_ shows a significant higher value in WG than DF [very large effect], shows a significant higher value in ST than DF [nearly perfect effect], and presents a significant higher value in WG than MF [large effect]. The TS_TD_ shows a significant higher value in MF than DF [large effect].

The TM_HSRD_ shows a significant higher value in WG than DF [large effect], presents a significant higher value in ST than DF [large effect], shows a significant higher value in WG than MF [very large effect], and shows a significant higher value in ST than MF [very large effect].

Finally, the TM_SpD_ presents a significant higher value in WG than DF [nearly perfect effect].

**Figure 5:**
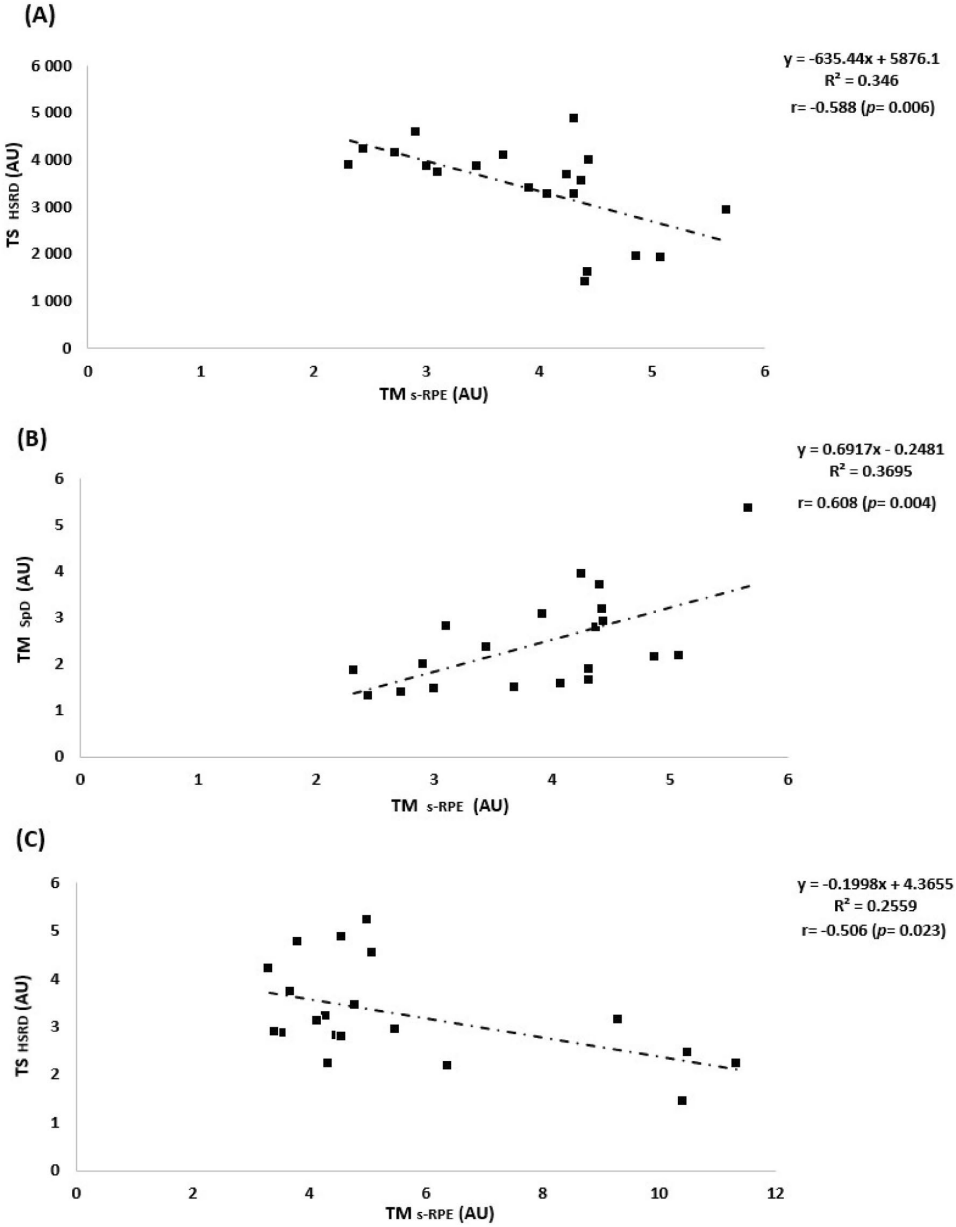
Pearson correlations in Early-season between TS HSRD and TM S-RPE (**A**); between TS SpD and TM s-RPE (**B**) and in Mid-season between TM HSRD and TM s-RPE (**C**).

Table 4 shows the correlation coefficient of all measures in the study for the team. In early-season, two positive correlations were denoted between: TM_TD_ and TM_s-RPE;_ TM_SpD_ and TM_s-RPE._ Two negative correlations were also denoted between: TS_HSDR_ and TM_s-RPE;_ TS_HSDR_ and TS_s-RPE._ In mid-season, one positive correlation (between TS_HSRD_ and TS_s-RPE_) and three negative correlations were denoted between: TM_SpD and_ TM_s-RPE;_ TM_TD_ and TS_s-RPE;_ TM_SpD_ and TS_s-RPE._ The correlations with large effects are presented in Fig. 5.

**Table 4:** Correlation analysis between external and internal load measures during the three periods of the in-season by the overall team.

Measures	TM_s-RPE_ (AU)	TS_s-RPE_ (AU)
**Early-season**		
TM_TD_ (AU)	0.474*	0.243
TS_TD_ (AU)	− 0.035	0.071
TM_HSRD_ (AU)	0.403	0.032
TS_HSRD_ (AU)	− **0.588#**	− 0.464*
TM_SpD_ (AU)	**0.608#**	0.342
TS_SpD_ (AU)	− 0.047	− 0.028
**Mid-season**		
TM_TD_ (AU)	− 0.338	− 0.463*
TS_TD_ (AU)	− 0.441	− 0.405
TM_HSRD_ (AU)	− 0.013	− 0.031
TS_HSRD_ (AU)	0.282	0.476*
TM_SpD_ (AU)	− **0.506#**	− 0.486*
TS_SpD_ (AU)	− 0.295	− 0.044
**End-season**		
TM_TD_ (AU)	0.069	− 0.313
TS_TD_ (AU)	0.333	0.190
TM_HSRD_ (AU)	− 0.030	− 0.334
TS_HSRD_ (AU)	0.084	0.027
TM_SpD_ (AU)	0.103	− 0.275
TS_SpD_ (AU)	0.115	0.040

Significant differences are highlighted in bold (*p* ≤ 0.05).

*AU* arbitrary units, *TM* training monotony, *TS* training strain, *s-RPE* session rated perceived exertion, *TD* total distance, *HSRD* high‐speed running distance, *SpD* sprint distance.

*moderate effect; #, large effect.

## Discussion

The aims of this study were to investigate the variations in internal and external workload measures of TM and TS in professional male soccer players according to periods of the season and playing positions, and to determine the associations between the same internal and external workload measures of TM and TS. The results revealed that regardless of measure, the highest mean TM and TS scores were observed in mid-season or end-season. This result contrasts with the findings of Fessi et al.^35^. They analyze the weekly variations of training-related monotony and strain in professional soccer players and found significantly higher scores in TM and TS during pre-season when compared to in-season. On the other side, results of the present study seem to be in line with the findings of Clemente et al.^22^. Those authors monitored the training load variables of professional soccer players across a 10-week period and found highest values of TM in pre-season and highest values of TS in early competitive season. Lastly, the results found by Oliveira et al.^7^ did not find significant differences across 10 mesocycles of the in-season period. As stated in the experimental design, coaches were responsible for training plan during the full-season which may help to explain the inconsistent results when analyzing other studies^22,35^ since different coaches may have different philosophies for training. Nonetheless, our study highlights the importance of quantifying load through the full-season to better understand the intensity variations of all players.

The results on the position-related differences in TM showed greater values for wingers and strikers which is not consistent with a previous study^7^ that showed a w-shape variation across 10 mesocycles from the in-season for all positions considering TM of HSRD (> 19 km/h) while the remaining TM values calculated through total distance or session rated perceived exertion were similar for all positions.

When TD and SpD were considered, midfielders exhibited greater TS scores. Contrasting results were obtained in a recent study^6^ that examine the differences between playing positions for TM and TS in professional players. They found no significant difference for both measures between positions. In another recent similar study^10^ weekly variations of external training loads throughout a professional soccer season were studied. While significantly greater TS values were reported for wingers and central defenders, no significant differences were found for TM between positions. Additionally, a recent study^7^ did not find such results. They found a tendency of higher values of TS of TD and HSRD (> 19 km/h) for wide defenders than central midfielders over the 10 mesocycles of the in-season.

Overall and considering TM, it was shows the uniformity of exercises during microcycles as well. These results warn the coaches that due to the training content of the game positions, they should pay more attention to the midfield positions (e.g., when compared to defenders and midfielders), which can keep them from the uniformity of the training. Whilst, if not observed, can lead to a decline in player performance and possibly detraining. While wingers and strikers, due to the nature of their playing positions in training and competitions, this problem is often not faced.

Correlation analysis for TM revealed that s-RPE is significantly associated with SpD in both early and mid-season. Results also showed a significant association between s-RPE and TD in early season. Regarding the TS, the results demonstrated that s-RPE is significantly associated with HSRD in early and mid-season. Current literature provides limited evidence on the relationships between different load measures to estimate TM and TS. Nevertheless, this result is in agreement with the findings of a previous study^23^ that investigated the association between s-RPE and external training load measures. Supportively, a significant association was noted between s-RPE and HSRD for a group of soccer players competing in the English Premier League^36^. According to Nobari et al.^8^, increasing internal intensity (e.g., HR and RPE) is linked to higher TM and TS, implying that increasing external intensity raises rating perceived. According to a previous studies^9,37,38^, an increase in TM can lead to overtraining, which is one of the consequences of a not well-adjusted training plan and, as a result, it raises the internal intensity during training and competition.

The present study has several limitations that should be taken into account. Firstly, the study data were obtained from one soccer team and thus it was conducted on a small sample. Secondly, generalizability of the results is limited to male professional soccer players. Lastly, the study lacks information about the injury records of players during training and match play across the different periods of the season. Therefore, further examinations are warranted to analyze the relationships between training load indices to estimate monotony and strain and injury in larger group of male and female soccer players from different age categories and competitive levels. The final limitation of this study was the lack of internal and external load monitoring in resistance training and competition sessions which should be considered in future studies.

## Conclusion

This study is original in the sense that it provides information regarding the variations in various internal and external training load measures of TM and TS with respect to the period of the season and the positions of the players. Our findings highlighted that TM and TS of professional soccer players is sensitive to period of the season, player’s position, and the measure used to estimate training workloads. Therefore, coaching staff should take into account these variabilities in order to identify the training requirements of players.

## Data Availability

The datasets generated during and analyzed during the current study are available from the corresponding author on reasonable request.
